# TME8/375: The collaborative Interface: A seamless web in the medical field

**DOI:** 10.2196/jmir.1.suppl1.e115

**Published:** 1999-09-19

**Authors:** B Dardelet

**Affiliations:** ^1^CNET, DIH/UCE, Issy les MoulineauxFrance

**Keywords:** Telemedecine, France, Evolution, Hierarchies

## Abstract

Just as information technologies have impacted other industries, the health industry is now beginning to perceive the consequences of this evolution and its vulnerability. These changes have strengthened particular representations and understandings of medical practices and their achievements in the world. A new organization was promoted by the attempts of structural organizations, like the NHS or the State to rationalize the number of institutional hospitalizations and to favor other alternatives, such as home care. The trend to use collaborative techniques passed and gave way to sociological reflections on these new techniques, which merge both social practice and modern technology while erasing each others distinction and purpose. We call this alteration of identities, both physical and professional, a collaborative controversy. In this paper, we will present, through a clear cartography of French telemedicine projects, the collaborative controversy, providing both the conditions and actors as well as their various connections. We tried to understand the consequences by following selected working applications to evaluate the conditions of the emergence of these projects, the possible convergence regarding scale change, and on the typology of their actors. The choices we make today with these new technologies will have broad reverberations on how we perceive suffering, sickness and even death in our modern societies.

